# Emotion Regulation Processes Can Benefit Self-Regulated Learning in Classical Musicians

**DOI:** 10.3389/fpsyg.2020.568760

**Published:** 2020-11-10

**Authors:** Ugne Peistaraite, Terry Clark

**Affiliations:** ^1^Centre for Performance Science, Royal College of Music, London, United Kingdom; ^2^Faculty of Medicine, Imperial College London, London, United Kingdom

**Keywords:** emotion regulation, self-regulated learning, reappraisal, musicians, performance training

## Abstract

Self-regulated learning (SRL) is the degree to which students are metacognitively, motivationally, and behaviourally active participants in their own learning process. It involves the self-regulation of cognitive, behavioural, and affective processes. SRL holds significant potential for enhancing practise and achievement. Although affect is acknowledged as one of the three fundamental processes in SRL, there is limited research investigating it. However, emotions have been found to influence SRL efficiency while emotion regulation (ER) can impact learning outcomes. Thus, this study sought to investigate how ER processes relate to SRL among professional musicians who perform Western classical music. Four forms of regulation (reappraisal, suppression, rumination, repression) were examined in relation to the SRL three-phase model. Professional musicians (*N* = 334) of 39 nationalities (age: 18–66; [*M* = 28]; female = 215; male = 119) completed a survey comprising the Self-Regulated Learning in Music Questionnaire, the Emotion Regulation Questionnaire, and demographic items. A significant positive correlation emerged between SRL and reappraisal, and significant negative correlations emerged between SRL and the other three processes. Further multiple linear regression analysis revealed that reappraisal, practise hours, and expertise accounted for 26% of the variance in SRL. Finally, a factorial (2 × 2 × 2) ANOVA yielded significant group differences on ER as a function of gender, expertise, and occupation. Results suggest that reappraisal can enhance the use of SRL in musicians, thus highlighting the potential utility in considering ER as part of SRL. These results suggest that by including training on emotion regulation strategies within musicians’ educational institutions and workplaces, efficiency and engagement in SRL can be enhanced. This could produce more effective learning strategies and outcomes, together with higher musical achievements.

## Introduction

The domain of music performance is a demonstrative environment in which to study emotions in learning. The process of preparing for a Western classical music performance naturally creates an environment filled with strong emotions which otherwise would be difficult to produce. Beyond expectations of expressivity, a performer may anticipate failure or success related to the real-time observation of an audience with a cultural expectation of no mistakes (Thompson, 2007). Furthermore, success depends on a series of interrelated factors, such as the listener’s affinity for the repertoire (Thompson, 2007), perception of the performer’s behaviour (Waddell and Williamon, 2017) and attractiveness (Wapnick et al., 2000; Griffiths, 2010), acoustics, and stereotyping (Davidson and Edgar, 2003). These factors can bring incredibly intense emotions not only when performing but during the preparation phase as well (Jones, 2012).

Nevertheless, until now, research in Western classical music performance has primarily focused on emotions experienced within performance situations, particularly stress, stage fright, and music performance anxiety (e.g., Kenny, 2005; Biasutti and Concina, 2014), as well as motivation (McPherson et al., 2018). To the authors’ knowledge, no previous research has looked at the role that emotions have in relation to the quality of musicians’ individual practise, which holds significant importance for the quality of performance (Williamon and Valentine, 2000; Jorgensen, 2004). While the role of emotion within musicians’ learning and practise settings is yet to be explored, research in educational psychology has revealed that students in learning settings “experience a rich diversity of emotions … [which] are significantly related to students motivation, learning strategies, cognitive resources, self-regulation, and academic achievement” (Pekrun et al., 2002, p. 91).

*Emotion* is a cognitively appraised conscious or unconscious response to an event, which “triggers a cascade of response tendencies manifest across loosely coupled component systems, such as subjective experience, facial expression, cognitive processing and physiological changes” (Fredrickson, 2001, p. 218). Some researchers also add the importance of behaviour as a part of emotional response (i.e., action tendencies; Gross, 1998). A similar event can evoke disparate emotions in different people, thus resulting in distinctive behaviours within learning processes. For example, take in comparison two classical pianists who receive negative feedback from their teachers. One starts feeling very anxious about an upcoming performance. She goes to a practise room to solve problems marked by her teacher but instead of practising she bursts into tears, becomes tense, struggles to concentrate, thus ends practise, and heads home to recover. Another student may interpret and appraise the same feedback as a chance to improve her playing for the upcoming performance, thus will feel determined and energised, and go directly to the practise room to fix all issues raised by a teacher.

Lazarus’ *Cognitive–Motivational–Relational-Theory* (CMRT; 2000) is one model that seeks to explain why people might respond to a similar emotion-evoking event so differently. According to CMRT (Lazarus, 2000), a person’s emotional experience depends on how an event or stimuli is appraised in relation to its positively or negatively significant meaning for one’s personal well-being goals. Appraisal, coping, and motivation form the meaning for each discrete emotion (so called *core relational themes*; Lazarus, 2000), which describe person–environment relationships (for example, anger—a demeaning offence against me and mine; hope—fearing the worst but yearning for better, and believing that improvement is possible; for more examples see Lazarus, 2000, p. 234). The relational meaning a person forms for each discrete emotion helps to define interpersonal and intrapersonal differences of emotional life. Furthermore, emotions may be transformed by changing the meaning of person–environment relationships or the environment itself.

*Cognitive–Motivational–Relational-Theory* (Lazarus, 2000) provides a clear illustration of how complex the emotion generation process is and, most importantly, explains the central role of cognition within emotions (Jones, 2012). This is necessary for understanding how emotions can be regulated in learning settings, as it is possible to change an emotional response by changing a learner’s thoughts. Although research in educational psychology has mainly focused on test anxiety (Zeidner, 2007; Ben-Eliyahu and Linnenbrink-Garcia, 2013), there is a growing body of research examining a broader range of emotions that emerge within educational settings (e.g., Schutz et al., 2006; Linnenbrink, 2007; Dettmers et al., 2011; D’Mello and Graesser, 2012; Goetz et al., 2012; Boekaerts and Pekrun, 2016). In today’s society, education is one of the most important long-term events throughout a person’s life span, contributing greatly to potential career success and quality of life. Thus, it may be assumed that many personal goals concerned with personal wellbeing stem from education. As described in CMRT (Lazarus, 2000), the higher the significance of a goal for a person’s well-being, the more intense the emotions that may be experienced in relation to it. Therefore, educational environments may be a boiling kettle of emotions. Recent research supports this assumption and is discussed next (e.g., Efklides, 2014; Boekaerts and Pekrun, 2016; Goetz et al., 2016; Hall et al., 2016).

Pekrun et al. (2002) investigated students’ emotions in learning settings through five qualitative, seven cross-sectional, three longitudinal, and one diary study using samples of university and school students. Although anxiety outweighed the reported frequency over other emotions (15–25% of all reported emotions), positive emotions, such as enjoyment of learning, hope, pride, and relief, were reported as often as negative emotions. Aside from anxiety, the other most often reported negative emotions were anger, boredom, and shame. Anxiety was mentioned not only in relation to taking tests but also for learning individually and in class. This demonstrates that learners experience a wide variety of emotions within their educational settings. Thus, it can be inferred that limiting the range of emotions examined within a study, and isolating them due to reasons of theoretical application, might exclude an important part of emotional experiences. Crucially for the present study, these discrete emotions were reported to interact with processes of self-regulated learning, which holds a significant potential for enhancing Western classical musicians’ individual practise and achievement.

In Western classical music performance, which is the domain of focus of this study, musicians spend a significant number of hours learning a piece and perfecting their performance skills alone (McPherson et al., 2018). This is very different from other performance domains, such as dance, sports, and theatre, where coaches and mentors not only develop an individually tailored training plan but also observe and monitor it on a daily basis. Nevertheless, a music student’s time spent alone with their instrument is crucial as this is when they develop and refine the requisite skills (Jorgensen, 2004). However, it has been demonstrated that during instrumental lessons teachers or mentors focus mainly on technical and interpretative nuances in music and give very little, if any, advice on effective practise strategies and how to achieve these musical nuances discussed in a lesson (Creech and Gaunt, 2012). As musicians practise and continue learning throughout their career lifespan, with experience they develop more efficient individual practise skills (McPherson and Renwick, 2001; Miksza, 2011). However, that is often attained through a trial and error approach and comes with a price of time. For these and other reasons, it is self-evident that self-regulation of one’s own learning process is of paramount importance for classical musicians. The psychological model of self-regulated learning (Zimmerman, 2002) has been demonstrated to be successfully applicable in the Western classical music performance domain and result in significant enhancement of musicians’ practise and achievement (McPherson et al., 2018).

**FIGURE 1:**
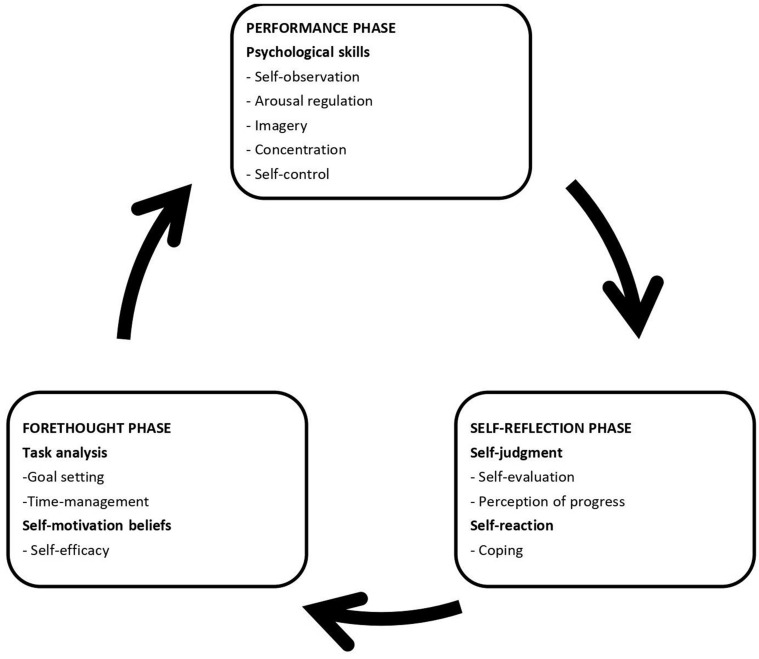
Zimmerman’s 3-phase self-regulated learning cyclical model adapted for music by Hatfield et al. (2017).

*Self-Regulated Learning* (SRL) is the degree to which learners are metacognitively, motivationally, and behaviourally active participants in their own learning process (Zimmerman, 1986). It involves the self-regulation of cognitive (thinking), behavioural (acting), and affective (feeling) processes (McPherson et al., 2018). Metacognitive processes involve planning, goal setting, self-monitoring, and self-evaluation; motivational processes involve self-efficacy, self-attributions, and intrinsic interest in the task; and behavioural processes involve selecting, structuring, and creating environments that optimise learning. These processes form a self-regulated learning cycle that has three phases: *forethought*, *performance*, and *self-reflection phases* (see Figure 1; Zimmerman, 2002; Hatfield et al., 2017). This cycle happens every time learning takes place and every phase predicts the others. *The forethought phase* happens before actual efforts to learn take place. The purpose of this phase is to enhance those efforts by processes of task analysis, planning, and self-motivation beliefs. This then informs *the performance phase* where the actual learning efforts occur. Its purpose is to improve action and self-monitoring through self-control and self-observation processes. After the performance phase follows *the self-reflection phase* in which the learner self-assesses their learning process to inform the next forethought phase through self-judgement and self-reaction processes (Zimmerman and Schunk, 2011).

The most relevant research in the classical music performance domain has mainly focused on behavioural, motivational, and cognitive processes when investigating SRL in musicians (McPherson et al., 2017). Very little, if any, research exists on the role of affective processes within music-specific SRL research (McPherson et al., 2018). The only evidence demonstrating the importance of affective background for musicians’ SRL is provided by Miksza (2009, 2011), where the role of impulsive and intuitive practise behaviour was examined among school and collegiate wind players. The findings revealed that students who were impulsive, reactive, and venturesome during their practise received lower performance scores.

However, research carried out in other academic performance domains has found emotions to be significantly related to SRL processes (Pekrun et al., 2002). In this research, Pekrun et al. (2002) grouped emotions as either positive activating (e.g., happiness), positive deactivating (e.g., relaxation), negative activating (e.g., anger), and negative deactivating (e.g., boredom), while still investigating each emotion as distinct. Positive activating emotions (e.g., enjoyment) were found to enhance motivation, whereas negative deactivating emotions (e.g., hopelessness and boredom) were found to reduce it. Interestingly, positive deactivating emotions (e.g., relief and relaxation) were found to weaken motivation, as did negative deactivating emotions. Negative activating emotions (e.g., anger, anxiety, and shame), meanwhile, were found to reduce intrinsic motivation but increase extrinsic motivation when the emotion is task related. Positive emotions, except for relief, and with no difference between the activating and deactivating groups, were also found to facilitate the use of flexible, creative learning strategies (such as elaboration, organisation, critical evaluation, and metacognitive monitoring) and increase attention (Pekrun et al., 2002). Negative activating emotions, meanwhile, were related to the use of more rigid and less creative learning strategies (such as repetition) and task-irrelevant thinking. Webster and Hadwin (2015) also found that positive emotions were positive predictors and negative emotions were negative predictors of self-evaluation of goal attainment.

To summarise, when comparing emotional experiences in self-regulated and externally guided learning, positive emotions have been found to be related to students’ self-regulation, whereas negative emotions related to reliance on external guidance (Pekrun et al., 2002). Knowing that emotions are related to SRL and that impulsive musicians implement poor practise strategies resulting in lower performance outcomes, it is the interest of this research to take a further step and investigate whether the ability to regulate one’s emotions may contribute to SRL.

*Emotion Regulation* (ER) has been defined as “the ways individuals influence which emotions they have, when they have them, and how they experience and express these emotions” (Gross, 1999b, p. 557). It involves changes in one or more of experiential, behavioural, and physiological response systems and need not (but certainly can) involve attempts to change the subjective experience of an emotion. ER can happen before the emotion arises, at the emotion-evoking event, or after the emotion has arisen. Emotions can be regulated to be increased or decreased depending on the emotion regulatory goals. Regulation before an emotion is triggered can be achieved by selecting the emotion-evoking situation, modifying that situation, deploying attention, and altering the meaning of the event (cognitive change). Regulation after an emotion has been triggered can happen only by regulating the emotionally expressive response.

There is little research that has examined the relationship between ER and SRL. However, Webster and Hadwin (2015) looked at undergraduate students’ self-evaluations of goal attainment, emotion intensity ratings, and open-ended descriptions of ER strategies and found that students tended to use a variety of ER strategies depending on the course. Ben-Eliyahu and Linnenbrink-Garcia (2013) used the Emotion Regulation Questionnaire (comprising of Nolen-Hoeksema et al., 1993; Gross and John, 2003) to investigate how undergraduate students regulate their emotions in their favourite and least favourite courses and found that students regulate their emotions more in their preferred courses. Finally, there is evidence from intervention studies that students who use more adaptive ER attain higher achievements (Punmongkol, 2009; Tzohar-Rozen and Kramarski, 2013). These findings highlight the potential utility in considering ER as part of SRL and reveal the importance of an emotional background when investigating resources of self-regulation.

Individuals differ in both which emotions they experience and in the ways they regulate them (Gross, 1999a). The most commonly studied ER processes are reappraisal, suppression, rumination, and repression (Gross, 1999a; Aldao et al., 2010; Ben-Eliyahu and Linnenbrink-Garcia, 2013).

*Reappraisal* involves modifying the way a situation is evaluated (Gross, 2015a). By reinterpreting emotion-evoking situations, the emotional experience can be changed. It is an adaptive form of ER which occurs before experiencing the emotion through attention deployment, cognitive change, or reappraisal of the emotional stimulus and involves cognitive processes to change the meaning of the emotion-evoking event (Gross and John, 2003; Ben-Eliyahu and Linnenbrink-Garcia, 2013). Reappraisers tend to approach stressful situations more positively and are more capable of improving a negative mood (Gross and John, 2003). A greater use of reappraisal is related to greater experience of positive emotions, lesser experience and expression of negative emotions, fewer depressive symptoms, and greater self-esteem (Gross and John, 2003). Cognitive reappraisal has been found to be one of the most effective ways of regulating negative emotions (Gross and Thompson, 2007).

Strain and D’Mello (2015) conducted an intervention study and found that adult learners achieved better scores than a control group who received no intervention when using cognitive reappraisal. Reappraising learners not only experienced more positive emotions but, more importantly, experienced activating positive emotions (i.e., alertness and engagement) which correlate positively with learning outcomes. The results were significant not only among those who reappraised constantly but also among those who reappraised less frequently as well. This indicates that learners can benefit even with minimal reappraisal. Ben-Eliyahu and Linnenbrink-Garcia (2013) found that students tend to use more reappraisal and experience more positive emotions in their favourite courses. Moreover, they also found that reappraisal benefited learning even in the courses deemed least favourite.

*Suppression* involves inhibiting emotion-expressive behaviour. Suppression intervenes in the emotion-generative process rather late and can only modify an individual’s expressive behaviour but not their emotional experience. While the use of suppression is very common in social situations in the Western world, greater use of this strategy may result in less positive emotion experience and expression, greater negative emotion experience, including painful feelings of inauthenticity, lower self-esteem, and higher levels of depressive symptoms (Gross and John, 2003). In learning, Ben-Eliyahu and Linnenbrink-Garcia (2013) found that suppression positively predicted maladaptive forms of coping (i.e., venting). Moreover, it related negatively to positive activating emotions (i.e., excitement) in favourite courses but positively related to positive deactivating emotions (e.g., relaxation) in least favourite courses. At this point, it is important to remember that positive activating emotions are found to be most beneficial for SRL, while positive deactivating emotions showed the opposite effect (i.e., Pekrun et al., 2002).

*Rumination* involves dwelling or focusing on experienced emotions. It involves attentional focus, relentless thinking, and behavioural styles on feelings and their consequences (Gross, 1999a, b). This mainly concerns negative emotional experiences: dwelling on problems, focusing on tiredness, and worrying about things that cannot be changed anymore. Rumination is conscious, effortful, and controlled, and, although it seems to be aimed at reducing negative feelings, it usually results in the opposite effect (Gross, 1999a). Suppression is often accompanied by rumination. Greater use of rumination has been found to be associated with a greater likelihood of developing depressive symptoms (Just and Alloy, 1997).

In learning, Ben-Eliyahu and Linnenbrink-Garcia (2013) found that rumination positively related with the participants’ negative emotions in both their favourite and least favourite courses and negatively related to positive emotions in their least favourite courses. Unsurprisingly, rumination is also considered to be a maladaptive form of ER in learning as the continuous thinking on emotional experiences draws the learners’ attention away from their learning processes.

*Repression* involves an automatic and unconscious attentional defence against unpleasant stimuli. Repressors can be recognised by very low scores in anxiety measures but higher measures in physiological arousal (in comparison to other people scoring low in anxiety) and very high scores in defensiveness (Gross, 1999b). Repressors also tend to be less expressive behaviourally, though this might change when subjects do not know they are being observed. Interestingly though, the repressor’s capacity for negative emotions is not diminished, although there is an absence of secondary emotions that usually follow an emotion experience. Unfortunately, to the authors’ knowledge, there is a lack of research that has explored the effects of repression on learning settings.

As evidenced by the educational psychology research reviewed above, emotions play a significant role in SRL. Moreover, although there remains limited evidence, ER processes have been demonstrated to have an influence on SRL. However, although the importance of SRL is greatly recognised within music, the role of emotions within it remains under-investigated at the moment. Instead, research has mainly focused on emotions when performing or in relation to motivation.

Following the need to investigate the role of emotions within SRL processes among musicians and building upon prior research which suggests that ER is part of SRL, the aim of this study is to understand the role of ER processes and their relationship to the ability to self-regulate one’s learning among Western classical musicians (by which is meant a musician of any nationality performing Western classical music). Therefore, this study is guided by the following question: “How do emotion regulation processes relate to self-regulated learning in musicians who perform Western classical music?” The hypothesis is that reappraisal relates positively to the use of self-regulated learning, whereas suppression, rumination, and repression relate negatively. In order to fully answer the research question, the following sub-hypotheses and research sub-question need to be addressed: (H1) “Emotion regulation processes relate to self-regulated learning in musicians.”; (H2) “Emotion regulation processes predict higher use of SRL.”; (RQ3) “Are there any group differences in these emotion regulatory processes regarding gender, level of expertise, or type of main occupation?”.

## Materials and Methods

### Participants

Classical musicians continue learning new repertoire throughout their whole career, therefore both an aspiring music performance student and an expert musician can and do engage in SRL (McPherson et al., 2018). As the quality of SRL and engagement with its processes increases with expertise, it is a common practise in the music performance domain to investigate how experts practise as to then compare and transfer this knowledge to less experienced musicians (McPherson and Renwick, 2001; Miksza, 2011). For this reason, musicians of any nationality and whose main income comes from a Western classical music occupation or is studying Western classical music performance as a main subject at a tertiary level were invited to participate in the study. A total of 537 professional Western classical musicians participated in the survey. Of the initial 537 participants, 203 participants (38%) were excluded as they failed to meet participation requirements or missed out an item in any of the measurements. This left a total of 334 (female = 215 [64%], male = 119 [36%]) respondents from 36 countries and of 39 nationalities that were used for data analysis (see Appendix 1, Figures A1.1, 1.2 within the Supplementary Materials for this article). Ages ranged from 18 to 66 years (*M* = 27.68, *SD* = 9.78), and years of experience in the music performance domain ranged from 3 to 59 years (*M* = 19.46, *SD* = 10.20). Of the sample, 56% (*n* = 187) were tertiary-level students and 44% (*n* = 147) were professional musicians. Making solo music was reported by 49% (*n* = 164) of the sample as their main activity, the other 51% (*n* = 170) reported making music in a group as their main activity. The distribution of the sample by instrumental groups was as follows: 36% (*n* = 121) played keyboard instruments, 29% (*n* = 98) played string instruments, 14% (*n* = 46) were singers, 14% (*n* = 45) played woodwind instruments, 6% (*n* = 19) played brass, and the remaining 1% of the sample did conducting, composition, or percussion (*n* = 5). The sample represented a broad spectrum of the overall population of Western classical musicians. A detailed demographic profile of the sample is presented in Appendix 1 within the Supplementary Materials for this article.

### Data Collection Tools

The complete cross-sectional survey comprised two questionnaires plus demographic items totaling 71 items (see Appendix 2 among the Supplementary Materials for this article).

#### The Self-Regulated Learning in Music Questionnaire

The Self-Regulated Learning in Music Questionnaire (SRLMQ; Hatfield et al., 2017) measures multidimensional self-regulated learning according to the constructs that represent Zimmerman’s (2002) 3-phase self-regulated learning model (see Figure 1). Hatfield et al. (2017) adopted this model to the context of higher music education. This questionnaire was used as it (1) was developed for musicians, (2) is relatively short (38 items) while still addressing the 3 phases of SRL, and (3) considers psychological skills.

The questionnaire comprises 12 scales (see Table 1). The forethought phase is represented by goal setting (α = 0.80, 6 items), self-efficacy (α = 0.77, 4 items), and time management (α = 0.73, 3 items) scales. The performance phase is represented by the psychological skills scales of self-observation (α = 0.76, 3 items), arousal-regulation (α = 0.58, 3 items), imagery (α = 0.87, 2 items), concentration (α = 0.64, 3 items), and self-control (α = 0.63, 3 items). Finally, the self-reflection phase includes self-evaluation (α = 0.75, 3 items), coping (α = 0.69, 3 items), and perception of progress (1 item) scales. Participants responded to all items using a 5-point Likert-type scale from 1 (Never/Strongly disagree) to 5 (Always/Strongly agree). In order to avoid repetitive questions for participants, all items were presented in a random order.

**TABLE 1:** The self-regulated Learning Questionnaire constructs (SRLQ, Hatfield et al., 2017).

Constructs	Items	α*	Item example
**Forethought phase**			
Goal setting	6	0.80	In relation to my long-term goals, I set specific short-term goals for my practise.
Self-efficacy	4	0.77	I can solve most problems if I invest the necessary effort.
Time management	3	0.73	I have a specific plan for how long each practise session should last.
**Performance phase**			
**Psychological skills:**			
Arousal-regulation	3	0.58	I often get overly tense during concerts and I am severely influenced by this.
Concentration	3	0.64	I easily get distracted while practising.
Self-control	3	0.63	I am tempted to hastily practise new pieces in the original tempo.
Self-observation	3	0.76	I check my accuracy while progressing through a practise task.
Imagery	2	0.87	I often use imagery in relationship to concerts and performances.
**Self-reflection phase**			
Coping	3	0.69	I think through past performance experiences to understand new practise ideas.
Perception of progress	1	–	I believe that my current progress reflects the amount of hours spent on practising.
Self-evaluation	3	0.75	I keep track of my progress over time.

*Alpha values from Hatfield et al. (2017).

#### The Emotion Regulation Questionnaire

As there were no emotion regulation questionnaires found that would measure the four emotion regulation processes which form the framework for this research, namely, reappraisal, suppression, rumination, and repression, the present questionnaire used for this study was combined from several sources and resulted in 19 items (see Table 2).

**TABLE 2:** The Emotion Regulation Questionnaire constructs.

ER process measured	Reappraisal	Suppression	Rumination	Repression
Items	6	4	4	5
α*	0.77	0.73	0.83	0.55
Item example	I control my emotions by changing the way I think about the situation I’m in.	I keep my emotions to myself.	I dwell upon the feelings the situation has evoked in me.	I never experience strong negative emotions.
Source	ERQ, Gross and John, 2003	ERQ, Gross and John, 2003	CERQ, Garnefski et al., 2002	Developed by Peistaraite for current study based on Gross, 1999b

*Alpha values from scale’s author.

To measure reappraisal and suppression, the complete Emotion Regulation Questionnaire developed by Gross and John (2003) (ERQ) was used. It comprises 10 items: 6 items for reappraisal (α = 0.77) and 4 items for suppression (α = 0.73). As requested by the authors in the ERQ manual, the order of the items was not changed, and participants rated each item using a 7-point Likert-type scale ranging from 1 (strongly disagree) to 7 (strongly agree). This ERQ by Gross and John (2003) comprised the first 10 items of the current Emotion Regulation Questionnaire used in this study.

The rumination scale was derived from the Cognitive Emotion Regulation Questionnaire (CERQ), developed by Garnefski et al. (2002) to measure cognitive coping strategies. The rumination scale has 4 items (complete scale, α = 0.83 [adult sample]). In order to avoid repetitive questions for participants, items were mixed with the repression scale developed by the first author of the current study.

The repression scale was developed by Peistaraite based upon Gross’s (1999b) description and major findings on this emotion regulatory process. The scale comprises 5 items (α = 0.55 [from the current study]). Participants responded to the rumination and repression items using a 7-point Likert-type scale, ranging from 1 (strongly disagree) to 7 (strongly agree). See Appendix 2 within the Supplementary Materials for the complete ERQ used in this study.

### Procedure

The project was approved by the Conservatoires UK Research Ethics Committee. The cross-sectional survey was delivered via SurveyMonkey.com, and convenience sampling was conducted via social media and email invitations.

After completing data collection and excluding unsuitable responses, statistical tests were used to check parametric assumptions and Cronbach’s alpha was used to estimate scale reliabilities. Then, to test the first sub-hypothesis (H1: Emotion regulation processes relate to self-regulated learning in musicians.), Pearson correlation tests were used. To further investigate the relationship and analyse the predictive power of ER to SRL, linear multiple regression was run to test sub-hypothesis 2 (H2: Emotion regulation processes predict higher use of SRL.). Finally, factorial (2 × 2 × 2) ANOVAs were used to investigate group differences for sub-question 3 (RQ3: Are there any group differences in these emotion regulatory processes regarding gender, level of expertise, or type of main occupation?).

## Results

Descriptive statistics for all variables are presented in Appendix 3 among the Supplementary Materials for this article. According to conservative criteria, the internal consistency of measures was satisfactory (Kerlinger, 1974). Some scales scored lower than in previous studies (Garnefski et al., 2002; Hatfield et al., 2017): self-efficacy (α = 0.63), time management (α = 0.62), self-control (α = 0.64), self-observation (α = 0.65), self-evaluation (α = 0.62), and rumination (α = 0.62). The repression scale (α = 0.55), which was developed for the current study, scored lowest and item-total statistics did not show any improvement if any of the items were removed. Item-total statistics for other scales, where removal of an item made an improvement, are in Appendix 4 among the Supplementary Materials for this article.

All variables were normally distributed, as skew values varied from 0.81 to −0.58. Moreover, histograms and Q–Q plots confirmed normality. Thus, parametric statistics were run. The results are presented in correspondence with hypotheses 1 and 2 and sub-question 3.

### Do Emotion Regulation Processes Relate to Self-Regulated Learning in Musicians?

Pearson correlations were conducted to examine relationships between the four emotion regulation processes and the total self-regulated learning score (see Table 3). To control for multiple comparisons across the four correlation tests, a Bonferroni correction was applied, thus the cutoff point for statistical significance was considered at 0.0125. A moderate significant positive correlation was found between reappraisal and the total SRL score (*r* = 0.38, *p* < 0.01), indicating that greater use of reappraisal related to greater use of SRL. A small significant negative correlation was found between the total SRL score and repression (*r* = −0.19, *p* < 0.01), which indicated that lesser use of repression related to greater use of SRL. No significant relationships were found between rumination or suppression and total SRL score.

**TABLE 3:** Pearson correlation between total SRL score and four ER processes.

Variable	Reappraisal	Suppression	Rumination	Repression
Total SRL score	***r* = 0.38**,	*r* = −0.12,	*r* = −0.05,	***r* = -0.19,**
	***p* < 0.001**	*p* = 0.02	*p* = 0.17	***p* < 0.001**
	*M* = 5.01,	*M* = 3.55,	*M* = 5.21,	*M* = 2.47,
	*SD* = 0.98	*SD* = 1.23	*SD* = 0.90	*SD* = 0.92

Table 4 illustrates a more detailed correlational analysis among ER and the SRL 3-phase model constructs. To control for multiple comparisons across the 48 correlation tests, a Bonferroni correction was applied, thus the cutoff point for statistical significance was considered at 0.001. Reappraisal correlated significantly positively with most of the constructs within the three phases of SRL. More specifically, within the forethought phase, small positive correlations were found between reappraisal and goal setting (*r* = 0.25, *p* < 0.001), self-efficacy (*r* = 0.27, *p* < 0.001), and time management (*r* = 0.16, *p* < 0.001). Within the performance phase, reappraisal showed small significant positive correlations with the total psychological skills scale (*r* = 0.27, *p* < 0.001) and, more specifically, with the subscales of imagery (*r* = 0.27, *p* < 0.001), concentration (*r* = 0.23, *p* < 0.001), and self-observation (*r* = 0.22, *p* < 0.001). The strongest positive relationships were found within the self-reflection phase. Reappraisal showed medium significant positive correlations with coping (*r* = 0.41, *p* < 0.001) and self-evaluation (*r* = 0.30, *p* < 0.001). These significant positive correlations indicated that greater use of reappraisal related to a greater use of these SRL constructs.

**TABLE 4:** Pearson correlation among ER and SRL 3-phase model’s constructs.

Variable	Reappraisal	Suppression	Rumination	Repression
**Forethought phase**				
Goal setting	0.25***	0.04	−0.00	−0.17
Self-efficacy	0.27***	−0.12	0.08	−0.22***
Time management	0.16***	0.04	−0.07	0.00
**Performance phase**				
*Psychological skills scale:*	0.27***	−*0.16*	−*0.15*	−0.20***
Arousal-regulation	0.12	−0.26***	−0.29***	−0.20***
Concentration	0.23***	0.08	−0.14	−0.13
Self-control	−0.02	−0.12	−0.18***	−0.15
Self-observation	0.22***	−0.01	0.07	−0.07
Imagery	0.27***	0.01	0.12	−0.06
**Self-reflection phase**				
Coping	0.41***	−0.01	0.07	−0.10
Perception of progress	0.16	−0.04	0.10	−0.04
Self-evaluation	0.30***	−0.06	−0.02	−0.10

***p < 0.001.

Furthermore, this more detailed correlational analysis yielded significant negative relationships between the three other emotion regulation processes (suppression, rumination, and repression) and the forethought and performance phases in the SRL 3-phase model constructs. Suppression showed a small significant negative correlation with arousal regulation (*r* = −0.26, *p* < 0.001) within the performance phase. Rumination showed small negative correlations with arousal-regulation (*r* = −0.29, *p* < 0.001) and self-control (*r* = −0.18, *p* < 0.001) within the performance phase too. Finally, repression showed a small negative significant correlation with self-efficacy (*r* = −0.22, *p* < 0.001) within the forethought phase. Also, repression showed small negative significant correlations with psychological skills (*r* = −0.20, *p* < 0.001) and arousal regulation (*r* = 0.20, *p* < 0.001) within the performance phase.

### To What Degree Do Emotion Regulation Processes Predict Higher Use of SRL?

Linear multiple regression was used to investigate the amount of variance in self-regulated learning scores that could be accounted for by the emotion regulation scales. The model included a total self-regulated learning score as the dependent/outcome variable and reappraisal, suppression, rumination, repression, practise hours, and level of expertise as independent/predictor variables. None of the correlation variables were above 0.60; therefore, substantial multicollinearity was not present (see Appendix 5 among the Supplementary Materials). All predictor variables were added by forced entry. The first block included reappraisal, suppression, rumination, and repression as predictor variables (measured on 7-point scales). The second block included two background variables: practise hours and level of expertise (student and professional categories coded, respectively). In the first model (see Table 5), reappraisal and repression were found to be significant predictors, explaining 19% of the variance in SRL (*R*^2^ = 0.19, *p* < 0.001). However, in the second model repression came out as a non-significant predictor. Both background variables contributed relatively equally to the model improving it significantly and together with reappraisal explained 26% of variance (*R*^2^ = 0.26, *p* < 0.001). All three predictors contributed significantly to the model. With other predictors held constant, each increase of 1 point in the reappraisal score predicted an additional 0.163 point increase in the SRL score (*b* = 0.163, β = 0.35, *p* < 0.001). Each increase of 1 h in practise hours per week predicted an additional 0.01 point increase in the SRL score (*b* = 0.010, β = 0.22, *p* < 0.001). Being in the professional category (in comparison to being in the student category) predicted an additional 0.19 point increase in the SRL score (*b* = 0.185, β = 0.20, *p* < 0.001).

**TABLE 5:** Regression models predicting Self-Regulated Learning total score.

Model predictor		*B*	Standard error	Standardised coefficients beta	*t*	*R*^2^	Adjusted *R*^2^	Change in *R*^2^
1	(Constant)	3.045	0.190		16.006***	0.19***	0.16	0.185***
	Reappraisal	0.175	0.023	0.381	7.545***			
	Suppression	−0.029	0.022	−0.079	−1.304			
	Repression	−0.066	0.029	−0.136	−2.243**			
	Rumination	-0.044	0.025	−0.089	−1.780			
2	(Constant)	2.727	0.190		14.34***	0.26***	0.25	0.077***
	Reappraisal	0.163	0.022	0.354	7.29***			
	Suppression	−0.029	0.021	−0.080	−1.37			
	Repression	−0.053	0.029	−0.110	−1.86			
	Rumination	−0.024	0.024	−0.049	−1.00			
	Practise per week (h)	0.010	0.045	0.222	4.57***			
	Level of musical expertise: student/professional	0.185	0.002	0.204	4.10***			

**p < 0.01, ***p < 0.001.

Casewise diagnostics confirmed residual statistics to represent the model accurately, and Cook’s values smaller than 0.04 indicated an absence of influence cases. Histograms and plots also confirmed homogeneity of variance and linearity. The VIF values ranged between 1.01 and 1.53, eigenvalues variance was distributed across different dimensions, and tolerance values ranged from 0.655 to 0.986, indicating there was no multi-collinearity in the model. Assumption of independent errors was confirmed by the Durbin–Watson test scoring at 1.91.

### Are There Any Group Differences in These Emotion Regulatory Processes Regarding Gender, Level of Expertise, or Type of Main Occupation?

The final phase of analysis sought to find out whether there were any group differences in the four emotion regulatory processes. To do that, a factorial (2 × 2 × 2) ANOVA was conducted for each of the emotion regulatory processes with gender, level of musical expertise (student or professional), and type of main occupation (solo or group music making) as independent variables and ER process (reappraisal, suppression, rumination, and repression) as the dependent variable (see Appendix 6 among the Supplementary Materials for descriptive and test statistics for all variables). To control for multiple comparisons across the four ANOVA tests, a Bonferroni correction was applied; thus, the cutoff point for statistical significance was considered at 0.0125. No significant group differences in reappraisal were found.

In suppression, the only significant difference found was a small main effect between the level of expertise [*F*_(__1_,_326__)_ = 7.849, *p* < 0.001, *η*^2^ = 0.02], where students (*M* = 3.72; *SD* = 1.27) used suppression more than professionals (*M* = 3.33; *SD* = 1.15). A similar trend in group differences was also found in repression. The only significant difference found was a small main effect between the level of expertise [*F*_(__1_,_326__)_ = 16.414, *p* < 0.0001, *η*^2^ = 0.05], where students (*M* = 2.65; *SD* = 0.97) used repression more than professionals (*M* = 2.23; *SD* = 0.81).

**FIGURE 2:**
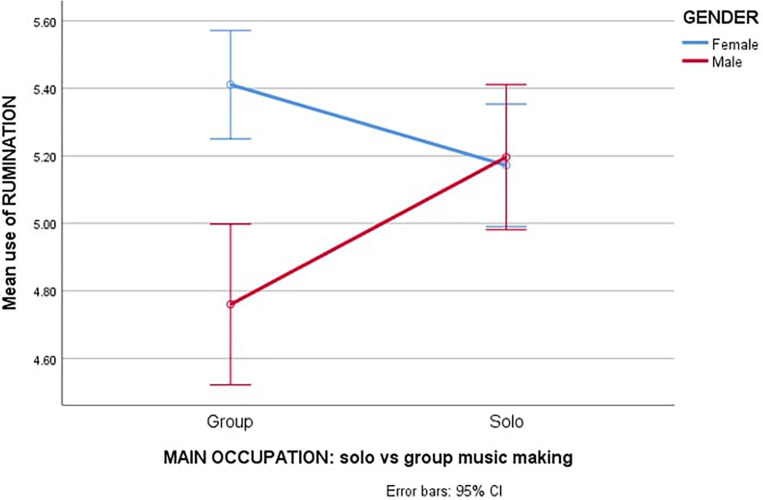
Estimated marginal means of rumination.

Lastly, in rumination (see Figure 2), a significant difference found was a small main effect between gender [*F*_(__1_,_326__)_ = 9.399, *p* < 0.001, *η*^2^ = 0.03], where females (*M* = 5.32; *SD* = 0.87) ruminated more than males (*M* = 5.01; *SD* = 0.93). Moreover, a significant small interaction was found between gender and type of main occupation (solo or group music making) [*F*_(__1_,_326__)_ = 10.917, *p* < 0.005, *η*^2^ = 0.03], in which the difference in rumination between male (*M* = 4.76, *SD* = 1.00) and female (*M* = 5.42, *SD* = 0.85) in group music making was much greater than in solo music making (male: *M* = 5.20; *SD* = 0.84; female: *M* = 5.21; *SD* = 0.88).

## Discussion

The purpose of the present research was to explore the relationships between four forms of emotion regulation (reappraisal, suppression, rumination, and repression) and SRL among musicians who perform Western classical music. A survey was completed by 334 professional musicians and tertiary-level students aspiring to become professionals and parametric statistical tests were used to analyse the data. The research hypotheses were partly supported (H1: Emotion regulation processes relate to self-regulated learning in musicians; H2: Emotion regulation processes predict higher use of SRL). As expected, higher use of reappraisal related to higher use of SRL. Lesser use of repression related to higher use of SRL. No significant overall relationships were found between the other two ER processes and the total SRL score. However, significant negative correlations were found between suppression and rumination and some of the constructs within the SRL 3-phase model. Furthermore, when considering the four ER processes simultaneously in the regression analysis, reappraisal predicted the use of SRL. Analysis of group differences in ER, answering research sub-question 3 (RQ3: Are there any group differences in these emotion regulatory processes regarding gender, level of expertise, or musical activity?) revealed that musicians are different from the general population in their use of ER which depends on their type of occupation (soloist or group musician) and professional experience rather than gender.

The positive and negative relationships observed between SRL and the four ER processes are not surprising, considering the previous findings on the general effects of those ER processes on individual functioning that were discussed earlier. In general, reappraisal is found to be adaptive and accommodating to an individual’s actions, resulting in the most positive experiences (Gross and John, 2003; Gross, 2014). Suppression, rumination, and repression, meanwhile, are related to negative experiences and are found to be cognitively taxing (Gross, 1999b; Jones, 2012). Current results are in line with Ben-Eliyahu and Linnenbrink-Garcia’s (2013) findings which demonstrated that reappraisal, when compared to suppression and rumination, supports SRL. Furthermore, reappraisal has been demonstrated to be useful as an intervention to improve comprehension in learning and related to higher learning achievements (Strain and D’Mello, 2015). As SRL has been found to enhance achievements (Miksza, 2015), the findings of the present study, together with earlier research, suggest that this relationship between reappraisal and achievement found by Strain and D’Mello (2015) can be explained by a higher use of SRL.

Reappraisal together with practise hours and level of expertise explained 26% of the variance in SRL tendencies (as a complete 3-phase cycle). The findings suggest that SRL might be enhanced by increasing the use of reappraisal together with the amount of practise and level of expertise. Ericsson et al. (1993)’s deliberate practise theory proposes that while accumulated practise hours account for becoming an expert and the quality of a musician’s performance, the quantity of practise has to be considered in line with the quality of practise. Given that deliberate practise is highly structured and effortful, Ericsson et al. (1993) caution that it is only possible to maintain the required level of focus and attention for so long, after which practise can become counter-productive. For example, the importance of quality of practise has been demonstrated by Williamon and Valentine (2000) who found that quantity of practise does not necessarily relate to the quality of performance. Therefore, encouraging students to practise more is not considered to be the recommendation made from this study. Instead, the implication of these results is to encourage and teach classical musicians to incorporate cognitive reappraisal into the planning, monitoring, executing, and evaluating of their learning while engaging in regular daily practise. Furthermore, these findings suggest that more experienced musicians may use reappraisal to a greater extent than less experienced musicians which might lead to greater self-regulated practise. The more detailed correlational analysis between reappraisal and SRL constructs within the 3-phase model (see Figure 1 for a visual representation of this model) facilitated delving deeper when analysing underpinning processes between ER and SRL.

Reappraisal correlated with most of the constructs within the SRL 3-phase model, except with arousal regulation, self-control, and perception of progress. Small correlations were found within the forethought phase, namely, goal setting, self-efficacy, and time management (smallest relation). Time management is not going to be discussed, as the relationship was so small (*r* = 0.16) that it may be trivial.

When trying to understand the positive association between reappraisal and goal-setting found in this study, the following factors could be taken into account. Goal-directed behaviour inherently involves emotion-eliciting situations (Saunders et al., 2015). If a particular situation has an influence on our goals, whether that is a positive or a negative influence, it will elicit an emotional reaction to it (Lazarus, 2000). That eliciting emotion can be regulated using different strategies at different points in time (Gross, 2015b). Depending on the ER process utilised, a person will end up with various emotional experiences and outcomes. As humans have an overall goal to feel good and in comfort (Saunders et al., 2015), someone who experiences strong negative emotions that they cannot handle every time their goal is challenged might avoid setting learning goals at all, or set very vague goals. On the other hand, let us think of someone who is capable of handling unpleasant experiences well in order to achieve their set goal to then be rewarded by an end goal of comfort. This latter person will feel more motivated to set goals and be better at persisting with a challenging task in order to complete that goal. Building upon this, reappraisal, being adaptive and often resulting in more positive and facilitative environmental experiences (Gross and John, 2003), could be used to facilitate the process of goal setting.

A somewhat similar situation can be proposed when interpreting the positive relationship between reappraisal and self-efficacy, which is a task-specific positive belief that one can execute the task successfully (Bandura, 1977). Self-efficacy feeds into increased motivation to set goals, as well as initiate and sustain self-regulatory efforts. According to Bandura (1986), emotions inform a person’s self-efficacy. Therefore, adaptive emotion regulation strategies, such as positive reappraisal, are thought to augment recognition of personal capabilities. Villavicencio and Bernardo (2016) found that positive emotions predict final grades, self-regulation, and self-efficacy, even after accounting for the variance explained by gender and anxiety. Through use of positive reappraisal, a more positive belief toward one’s self-efficacy can be enabled to deliver the intended SRL goals and exert more effort (Hanley et al., 2015). Furthermore, self-efficacy is influenced by a person’s past experiences, such as successes and failures when completing a similar task. In their intervention study, Hanley et al. (2015) found that positive reappraisal partly accounted for learners’ higher academic self-efficacy after experiencing academic failure. Given this, it would appear that positive reappraisal can be used to reframe past negative learning experiences by attributing it to one’s lack of skills rather than ability. This could mean that by engaging in sufficient practise to further develop one’s skills, another failure could be avoided. Additionally, personal resources could be reinterpreted as being sufficient to handle the task (McRae et al., 2012). Although more empirical evidence is needed to explain the underpinning processes between reappraisal and self-efficacy, these findings are in line with the intervention studies by Hanley et al. (2015) and Villani et al. (2017), where reappraisal was also found to be associated with higher self-efficacy.

Reappraisal also showed small correlations with performance skills in the performance phase. More specifically, reappraisal correlated with concentration, self-observation, and imagery. Emotions, especially acute ones, can be cognitively taxing and, thus, distracting. This can impair a person’s presence and make it difficult for them to concentrate on anything other than their emotional experience (McRae, 2016). Reappraisal has been demonstrated to successfully change disturbing experiences to more adaptive ones (Gross, 2015a) and, through this, accommodate concentration and self-observation. By eliminating distracting emotional experiences (either negative or positive), one should be able to focus more toward the task as well as self-observe the process of delivering the task.

The results of the current study indicate that learners who use reappraisal also use imagery more. It is difficult to say why this could be the case, as no previous studies investigating such a link were found. The two items in the SRL questionnaire (see Appendix 2 within the Supplementary Materials for this article) only identify if a person is using imagery in relation to practise and in relation to performance; the SRL questionnaire does not investigate what kind of imagery a person is using (e.g., to visualise a successful performance of a technically difficult passage, memorising a manuscript, imagining emotional expression and interpretation, etc.). Therefore, it is difficult to theorise what might be the underpinning processes between reappraisal and imagery. Future research could look into this link in more detail and investigate how different imagery strategies relate to reappraisal, as well as investigate the causational direction. It may be that, for example, musicians who are more in tune with their emotions engage in greater amounts of imagery practise or use a particular type of imagery more (e.g., imagining feeling good and not anxious when performing a piece). It can also be the case that imagery is used to reappraise a particular situation or resources related to emotional stimuli.

The strongest correlations were found between ER and the self-reflection phase of SRL. Reappraisal showed a moderate correlation with coping and self-evaluation. The self-reflection phase is commonly avoided by musicians (Waddell and Williamon, 2019). One of the reasons may be that self-reflection can bring unpleasant emotions of dissatisfaction or disappointment with the progress made. At a theoretical level, it can be expected that a person who is able to manage dissatisfaction by reappraising a situation more positively would be more inclined to engage in self-evaluation. Dweck (2017) described how two contrasting perceptions of learning progress, helpless-oriented and mastery-oriented, may lead to different engagement in the learning process. Applying this theory to musicians, helpless-oriented musicians would be expected to experience more negative emotions when faced with challenges and eventually give up (O’Neill and Sloboda, 1997). Instead of suppressing dissatisfaction with the progress made, or ruminating on it, one might reappraise it to be viewed as a learning and improvement opportunity, this way enabling a mastery-oriented mindset which leads to persistence and embracement of a learning challenge (Dweck, 2017).

The relationship with coping, meanwhile, is in line with previous research demonstrating that individuals who use adaptive ER strategies, like reappraisal, also use more adaptive coping styles (Moors et al., 2013). Nevertheless, Taylor and Wilson (2016) found that cognitive reappraisal significantly predicted future time perspective in relation to past goal failure and striving for new goals. In other words, after failing to meet goals, reappraisers invested more effort and planned more in a subsequent cycle of goal attainment. Finally, it is likely that reappraisal benefits self-reflection by helping a learner to select attitudes that would be directed to a lack of a skill or strategy rather than a lack of ability (Ben-Eliyahu and Linnenbrink-Garcia, 2013). While a lack of ability might hinder self-efficacy, attributing failure to a lack of skill or strategy could encourage a person to engage in more rigorously planned practise (Hatfield et al., 2017; Dweck, 2017). Therefore, these findings suggest that reappraisal might accommodate the self-reflection phase and adaptively inform the forthcoming forethought phase.

With regard to suppression, results showed that greater use of suppression was associated with decreased arousal regulation within the performance phase. Earlier research has demonstrated that suppressing emotional expression does not help to reduce the experience of an emotion (Gross and John, 2003). In fact, it results in a greater experience of negative emotions and lesser experience of positive emotions. Moreover, suppression is often followed by rumination, which augments and prolongs a negative experience as well as impairs concentration (Gross, 1999b). This might explain the negative correlation found between arousal regulation and suppression, implying that less use of suppression relates to better arousal regulation.

Rumination showed small correlations with arousal regulation and self-control within the performance phase of SRL. It is reasonable that rumination would be considered to be maladaptive when the actual learning takes place (performance phase) as the relentless overthinking about experienced feelings is cognitively taxing (Gross, 1999b). It may divert thought processes away from performing learning tasks by taxing cognitive resources and, by this, impairing self-control (Ben-Eliyahu and Linnenbrink-Garcia, 2013). Incessant dwelling on negative feelings, meanwhile, impairs arousal regulation by augmenting and prolonging negative emotions (Gross, 1999b). This explains how rumination could negatively affect the performance phase, as enhanced and prolonged focus on negative emotions will come at a price of self-control and impair arousal regulation.

Repression results are not discussed as correlations were small and the reliability of the scale was low (α = 0.55). However, findings are reported in the Result section and scale items can be found in Appendix 2 within the Supplementary Materials for this article in case of interest of replication. It is important to note though that repression, being an unconscious emotion regulatory process, might be the reason why the reliability of this scale was low, as it may be difficult to measure on self-report tools. Therefore, future research could consider alternative options for repression measurement.

### Group Differences in ER in Musicians

The last phase of analysis aimed to explore ER tendencies and profiles between different Western classical musician populations. Statistical group comparison tests were run to investigate for any group differences based on gender, level of expertise (student or professional), and type of musical activity (solo or group), while excluding ER’s relationship to SRL. While there were no group differences in reappraisal, which is in line with previous research (Gross and John, 2003; Ben-Eliyahu and Linnenbrink-Garcia, 2013), analysis revealed that Western classical musicians are different from the general population with regard to gender differences in suppression and rumination. Furthermore, students were found to use suppression more than professionals.

In suppression, which involves inhibiting emotional expression (but not the emotional experience), gender differences were not evident in this sample. The results are not consistent with earlier findings by Gross and John (2003) and Ben-Eliyahu and Linnenbrink-Garcia (2013) in which males were found to use suppression more than females. According to Gross and John (2003), the reason behind this gender difference is the traditional male upbringing of “boys don’t cry” where males are taught to suppress their emotional expression because showing it is generally perceived as a weakness. In music, though, emotions expressed through music are highly valued, expected, and even perceived as inevitable to enrich the performance and the listener’s experience (Juslin and Timmers, 2010). Therefore, musicians are constantly thinking and striving for how to make a musical piece more emotionally expressive (Woody and McPherson, 2012). On the other hand, in pursuit of delivering an expressive performance, due to Western classical cultural expectations of a confident and flawless performer, classical musicians, regardless of gender, aim to suppress signs of stress and music performance anxiety when performing (Woody and McPherson, 2012). This could explain why gender differences in suppression among classical musicians are not evident when compared to the general population.

With regard to the finding that student musicians suppress more than professionals, it is in line with previous research showing that individuals suppress their emotions in front of significant or dominant others (Gross and John, 2003). The tradition of instrumental/vocal teaching is predominantly teacher-directed (Gaunt, 2010). From this, it could be proposed that student musicians suppress more than professionals as they are surrounded by significant others, such as their instrumental mentors.

In rumination, findings are in line with previous research on general populations by Gross and John (2003) where females have been found to ruminate more than males. However, in the current study, such a situation was evident within group music making but the rumination gender difference decreased significantly within the soloist group (see Figure 2) where both men and women ruminated at about the same level. Ben-Eliyahu and Linnenbrink-Garcia (2013), who investigated how students use ER in learning settings, found that this gender difference was evident only in the participants’ least favourite courses when compared to their favourite. This might suggest that making music in a group is less enjoyed by musicians than being a soloist. One such reason might be the loss of autonomy in an ensemble, especially in a larger one, such as an orchestra (Davidson and King, 2004). The interpersonal dependency which can be caused by the loss of autonomy has been found to be linked to rumination (McBride and Bagby, 2006). Furthermore, rumination can then be augmented by co-rumination, which is defined as joint discussion and dwelling on negative feelings with others (Carlucci et al., 2018). The co-rumination with colleagues about the loss of autonomy, among other things, could also explain the difference in the rumination between group and solo classical musicians.

However, these explanations in group differences are based on theoretical reasoning, as empirical research on ER processes in classical musicians was not found. This study revealed that, interestingly, musicians’ use of ER differs depending on the type of occupation (soloist or group musician) and professional experience of the musician, rather than their gender. It could also be argued that, for example, a soloist needs to recruit different and more adaptive ER strategies than orchestra or choir musicians in order to sustain their career and successfully meet various challenges.

### Suggestions for Future Research

This study represents a starting point of an interrogation into the role of ER in this highly recognised niche of research on self-regulated learning, and in music performance science as well as education in general. The suggestions for future research follow two main streams: researching underpinning processes between reappraisal and SRL and investigating other ER processes that might play a role within SRL.

As reappraisal showed the most promising results by being the strongest predictor of SRL, future research could focus in this direction and investigate the causality, how reappraisal interacts with SRL, what processes underpin this interaction, and what potential reappraisal strategies might best enhance the use of SRL. There are endless ways to reappraise an emotional stimulus; one can adopt a different mindset, use optimistic beliefs, find meaning in their feelings, interpret a detached viewpoint, pretend it is not real, and others (Kross, 2015). While some of these strategies might be helpful, others might not, and it could depend on more detailed circumstances. Saunders et al. (2015) demonstrated how the successful downregulation of a negative emotion might actually result in reduced cognitive self-control. This may, for example, happen when a negative emotion, elicited due to incongruence with a learning goal (this way prompting greater exertion of cognitive self-control), is downregulated by reappraising the importance of the goal to achieve an overall goal of cognitive comfort. This suggests that reappraisal goals should align with learning goals for reappraisal to be helpful. Although reappraisal is most often used to decrease negative emotion, it can be used for both up- and downregulating negative or positive emotions (Ochsner and Gross, 2005). This highlights that it is not a down- or upregulation of negative or positive emotion itself that has an impact on SRL but rather an interaction with the SRL processes and reappraisal tactics used.

Microanalysis (McPherson et al., 2017) could be an excellent tool to investigate such interactions. Recorded practise sessions, participant interviews before and after the session supported by a video review, questionnaires, and practise diaries would allow researchers to better document emotional experiences and reappraisal strategies used within practise settings. Furthermore, reappraisal use can be trained and increased (Denny and Ochsner, 2014). In sports, the common strategies used by consultants to reappraise an event with athletes are didactic approaches, Socratic dialogue, self-analysis, reframing, cognitive paradox, and the use of storytelling, metaphors, and poetry (Jones, 2003). These strategies could be employed within musicians’ practise, too. As microanalysis can be used as an educational tool for participants, a researcher could prompt a participant on the use of reappraisal (McPherson et al., 2017).

On the contrary, there are cases when reappraisal might not be the most helpful option, such as when an emotion is sudden, perceptually based, or strong (McRae, 2016). Therefore, it is important to continue exploring the role of other ER strategies to better illuminate their role in SRL. One such strategy worth attention could be distraction, which is found to be more efficient in situations where an individual’s access to cognitive resources is compromised (McRae, 2016). Nevertheless, it would be useful to consider different ER strategies in relation to emotions as related and unrelated to learning, rather than positive or negative. As Saunders et al. (2015) suggest, a negative emotion elicited due to a risk of failing one’s learning goals might, in fact, encourage the learner to exert more effort in cognitive self-control. On the other hand, being very excited about an upcoming holiday, while a positive experience, might distract the learner from executing their learning plan. Future research could investigate how the efficiency of the utilisation of ER strategies might differ depending upon whether emotions are related or unrelated to learning.

Although the current study did not find strong significant relationships between the total SRL score and the four ER processes, it is still important to consider their role within SRL at this stage. With regard to suppression and rumination, it was expected that a greater negative correlation with the total SRL score would be found because it results in a greater and prolonged experience of negative emotions (Gross and John, 2003), which has been found to lead to a lesser use of SRL (Pekrun et al., 2002; Ben-Eliyahu and Linnenbrink-Garcia, 2013). There is evidence that students regulate their emotions somewhat differently depending on the context, subject, and also how much they enjoy the subject (Ben-Eliyahu and Linnenbrink-Garcia, 2013). Therefore, it is suggested that ER is context-specific. For example, a person who tends to suppress their emotions in front of other people might regulate them differently when alone in a practise room; they might reappraise those emotional experiences as unimportant and concentrate on practise tasks. The current study used general ER scales that were not tailored toward musicians’ practise settings. In order to better understand musicians’ ER processes in learning, it might be worth developing an ER questionnaire specifically for musicians’ practise. Furthermore, suppression did not show any correlation with coping within the self-reflection phase in SRL, which has been found in other studies (Gross, 1998; Butler et al., 2003; Gross and John, 2003; John and Gross, 2004). It is possible that the results differed because of the measures used. Earlier studies looked at maladaptive coping, whereas this study used a problem-solving coping scale, thus assessing different coping strategies. It may be the case that suppression is more clearly associated with maladaptive coping but generally unrelated to problem solving coping. Finally, with regard to repression, because it is an unconscious ER process and due to the absence of empirical evidence in the educational domain, this poses greater challenges of measurement. Future research should consider alternative options other than self-report that might be more suitable to measure it within learning environments.

More generally, this study demonstrated that Western Classical musicians differ from the general population in their use of ER processes. It is for future research to investigate classical musicians’ emotion regulatory profile and find out how musicians regulate their emotions, potential differences between different instrumental and occupational groups, and whether musicians’ ER is context specific. Understanding more about ER tendencies among Western classical musicians would inform other potential links, such as music performance anxiety, well-being, and coping with career challenges. It would also help to direct and choose appropriate research methods for SRL in relation to affective processes.

On a broader spectrum, the lack of even nationality distribution restricted this study from looking at cultural differences in ER, which have been found in previous studies by Gross and John (2003). Previous findings suggest that the effects of emotion regulation may be moderated by cultural values. For example, Butler et al. (2003) found that an individual holding Western-European values will have a greater negative experience with the use of suppression when compared to an individual holding Asian values. In relation to the current study, such differences might have skewed the relationship between ER and SRL due to the wide spread of nationalities represented within the participant sample. Future research could control for differences in cultural values when investigating the relationship between ER and SRL.

### Conclusion

The influential role of emotions and emotion regulation in relation to SRL processes and learning outcomes has been examined within academic performance domains yet remains under-researched in relation to Western classical musicians. The results of this study show that ER processes do have an impact on SRL in music as well. The current study demonstrated that by utilising adaptive ER strategies, such as reappraisal, a learner is more likely to engage in SRL processes. Although other ER strategies require further investigation, this study proposes that ER should be incorporated more broadly within the SRL model as emotional self-regulation, rather than considered only in relation to motivation. This would extend the model beyond the self-regulation of cognitive, behavioural, and motivational processes (Zimmerman, 2000; Ben-Eliyahu and Linnenbrink-Garcia, 2013; McPherson et al., 2018).

As reappraisal showed the most promising results, future research could investigate in more depth the underpinning processes and links between reappraisal and SRL, as well as what reappraisal strategies enable greatest engagement in SRL. Nevertheless, it would be important to continue investigating how other ER processes, such as distraction from emotional stimuli, may impact SRL. This would help produce a clearer picture of other potentially useful ER strategies. Finally, as musicians differed from the general population in terms of their use of ER, it is important to determine more thoroughly musicians’ emotion regulatory profile in order to be able to develop and apply ER interventions.

The main practical implication of this research is the benefits that could be obtained by incorporating reappraisal training into Western classical musicians’ educational programmes on SRL and learning environments in general. This could be done not only by educational institutions, such as music conservatoires but also by significant others, such as instrumental teachers and parents. Furthermore, as musicians continue learning throughout their career lifespan, delivering such training within working institutions, such as orchestras, could be of value as well. By acknowledging emotions in learning environments and reappraising them, efficiency and engagement in SRL can be enhanced, which stands to produce more effective learning strategies and outcomes, together with higher musical achievements.

## Data Availability Statement

The raw data supporting the conclusions of this article will be made available by the authors, without undue reservation.

## Ethics Statement

The studies involving human participants were reviewed and approved by CUK Research Ethics Committee. The patients/participants provided their written informed consent to participate in this study.

## Author Contributions

Both authors listed have made a substantial, direct and intellectual contribution to the work, and approved it for publication.

## Conflict of Interest

The authors declare that the research was conducted in the absence of any commercial or financial relationships that could be construed as a potential conflict of interest.
