# Supplementary material for: Emotion Regulation Processes Can Benefit Self-Regulated Learning in Classical Musicians
Source: Front Psychol. 2020 Nov 10;11:568760. doi: 10.3389/fpsyg.2020.568760 (PMC7683505; doi:10.3389/fpsyg.2020.568760)
Supplement: Supplementary file 1 [file Data_Sheet_1.docx]

Supplementary Material

# Supplementary Data

# APPENDIX 2: Online Survey Questionnaires

*It would be much appreciated if you could answer the questions as truthfully as possible. There are no right or wrong answers.*

**Demographics:**

- Your nationality:
- Country of your studies:
- Country of residence:
- Age:
- Gender:
  - Female
  - Male
  - Other:
- What is your main instrument?
- What is your primary musical genre?
  - Classical
  - Jazz
  - Pop
  - Folk
  - Other
- For how many years have you been playing/singing?
- For approximately how many hours do you usually practice per week?
- For approximately how many hours do you usually spend rehearsing with others per week?
- Your highest degree you hold or are currently studying:
  - Bachelor/Undergraduate
  - Master/Postgraduate
  - PhD/DMus/DPerf
- What best describes your musical activity?
  - Amateur
  - Student: secondary school
  - Student: undergraduate
  - Student: postgraduate
  - Professional (i.e. majority of income arises from music).
- If you are professional musician, working as:
  - Freelancer
  - Under a contract
  - Other:
- Main occupation (select as many as apply):
  - Orchestra
  - Choir
  - Teaching
  - Accompanying
  - Solo
  - Chamber
  - Other:

**SELF-REGULATED LEARNING IN MUSIC QUESTIONNAIRE***

For each question please answer using the following scale:

1 2 3 4 5

Never Seldom Sometimes Often Always

**Goal setting**

1. I always set concrete long-term goals for myself.

2. In relation to my long-term goals, I set specific short-term goals for my practice.

3. On a daily or weekly basis, I set very specific goals for myself that guide what I do.

4. I set very specific goals and know what it takes to reach them.

5. I clearly plan my course of action to solve a problem in my instrumental practice.

6. I develop a specific plan for the solution of a problem in my practice.

**Self-efficacy**

7. I strongly believe that I have what it takes to accomplish what I start working on.

8. I can solve most problems if I invest the necessary effort.

9. I always manage to solve difficult problems if I try hard enough.

10. I believe that I am able to become more consistent and goal-directed in my instrumental practice.

**Time-management**

11. I plan how long I should practice before taking brakes.

12. I am currently not managing my time of practice.

13. I have a specific plan for how long each practice session should last.

**Self-observation**

18. I check my accuracy while progressing through a practice task.

19. I observe my practice from an analytical perspective while practicing.

20. I check how well I am doing when I solve instrumental practice tasks.

**Arousal-regulation**

21. I often get overly tense during concerts and I am severely influenced by this.

22. I usually communicate negatively with myself while practicing.

23. I think about and imagine what will happen if I fail or screw up before concerts.

**Imagery**

24. I often use imagery in relation to instrumental practice.

25. I often use imagery in relation to concerts and performances.

**Concentration**

26. It is easy for me to direct my attention and focus towards what I am practicing

27. It is easy for me to keep distracting thoughts from interfering with my instrumental practice.

28. I easily get distracted while practicing.

**Self-control**

29. I tend to lose focus towards task while practicing due to a desire to master the task immediately.

30. I am tempted to hastily practice new pieces in the original tempo.

31. I am unfortunately not consistent enough with my instrumental practice.

**Self-evaluation**

32. I keep track of my progress over time.

33. When having practiced something during longer periods, I look back to see if I did the right procedures.

34. I am generally good at evaluating my instrumental practice and finding adequate solutions.

**Coping**

35. When things turn out badly during concerts, I try to think about how I can do things better next time.

36. I think through past performance experiences to understand new practice ideas.

37. When I´m not achieving the desired results, I carefully search for plausible reasons -

that leads to new adequate goals.

**Perception of progress**

38. I believe that my current progress reflects the amount of hours spent on practicing.

********Questionnaire developed by Hatfield, J. L., Halvari, H., and Lemyre, P. M. (2017), Instrumental practice in the contemporary music academy: A three-phase cycle of Self-Regulated Learning in music students. Musicae Scientiae 2017, Vol. 21(3), p. 316 –337.*

**EMOTION REGULATION QUESTIONNAIRE*:**

For each question please answer using the following scale:

1 2 3 4 5 6 7

(strongly disagree) (neutral) (strongly agree)

1. When I want to feel more positive emotion (such as joy or amusement), I change what I’m thinking about.

2. I keep my emotions to myself.

3. When I want to feel less negative emotion (such as sadness or anger), I change what I’m thinking about.

4. When I am feeling positive emotions, I am careful not to express them.

5. When I’m faced with a stressful situation, I make myself think about it in a way that helps me stay calm.

6. I control my emotions by not expressing them.

7. When I want to feel more positive emotion, I change the way I’m thinking about the situation.

8. I control my emotions by changing the way I think about the situation I’m in.

9. When I am feeling negative emotions, I make sure not to express them.

10. When I want to feel less negative emotion, I change the way I’m thinking about the situation.

11. When something unpleasant happens to me, I tend to act like nothing has happened, ignore that fact, and do other things.

12. I often think about how I feel about what I have experienced.

13. I never experience strong positive emotions.

14. I am preoccupied with what I think and feel about what I have experienced.

15. Others tell me I am an unemotional person, and I do not understand why.

16. I want to understand why I feel the way I do about what I have experienced.

17. I never experience strong negative emotions.

18. I dwell upon the feelings the situation has evoked in me.

19. I often do not realise how I am feeling until others point it out to me.

**Questions 1-10: Emotion Regulation Questionnaire (ERQ) by Gross, J. J., and John, O. P. (2003). Reappraisal Items (Cronbach’s alpha .77): 1, 3, 5, 7, 8, 10; Suppression Items (Cronbach’s alpha .73): 2, 4, 6, 9.*

*Questions 12, 14, 16, 18: extracted from Cognitive Emotion Regulation Questionnaire (CERQ) by Garnefski, N., Kraaij, V., and Spinhoven, P. (2002), Rumination scale (complete, Cronbach’s alpha .83 (adults)), questions: 3, 12, 21, 30.*

*Questions 11, 13, 15, 17, 19: repression items developed by Peistaraite based on Gross, J. J. (1999) Emotion Regulation: Past, Present, Future, Cognition and Emotion.*

# Supplementary Figures and Tables

## Supplementary Figures

# APPENDIX 1: A Detailed Demographic Profile of a Sample.


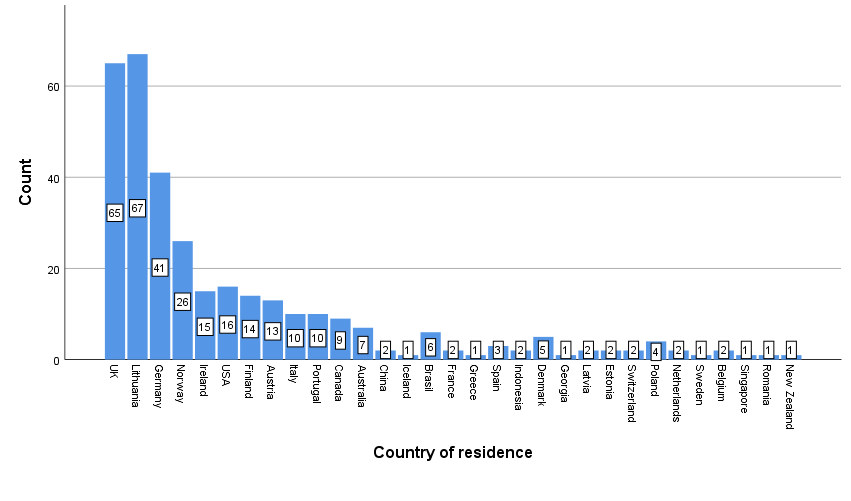


*Figure A1.1.* Sample frequencies according to a country of residence.


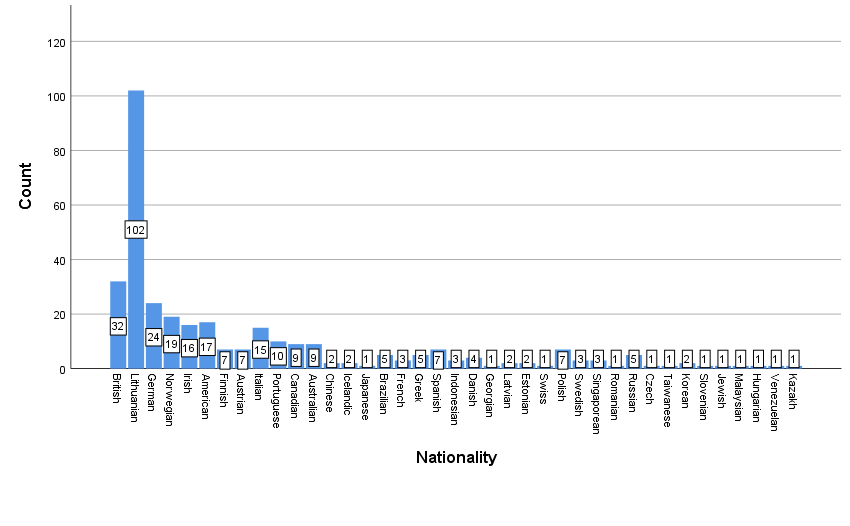


*Figure A1.2.* Sample frequencies according to a nationality.


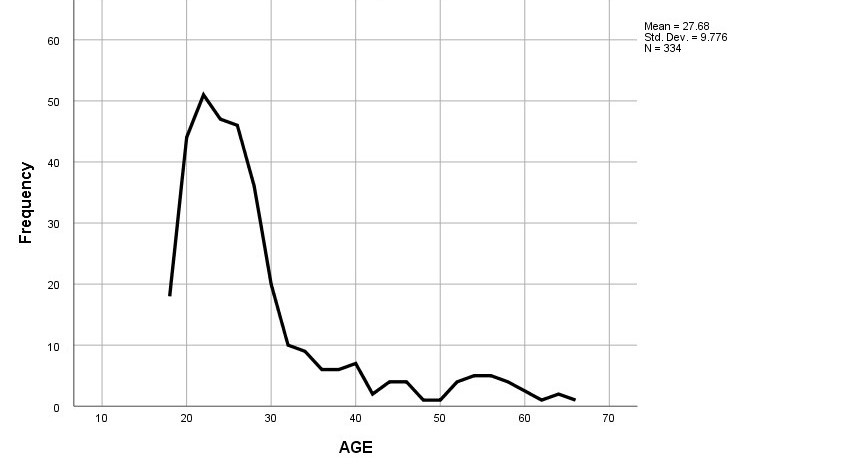


*Figure A1.3.* Sample frequencies according to age.


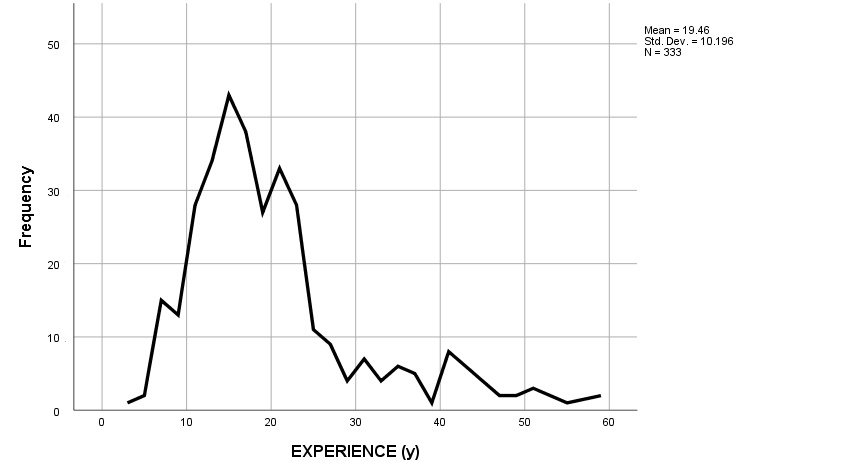


*Figure A1.4.* Sample frequencies according to experience (in years).


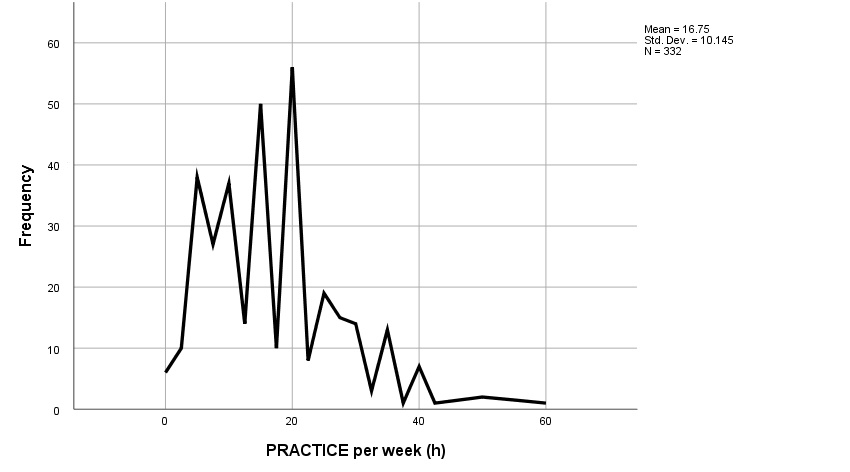


*Figure A1.5.* Sample frequencies according to accumulated practice hours per week.

## 2.2 Supplementary Tables

# APPENDIX 3: Descriptive statistics for all quantitative variables

*Table A3****.*** Descriptive statistics for all quantitative variables.

| *Variable* | *Items* | *α* | *M* | *SD* | *Skewness* | *Kurtosis* |
| --- | --- | --- | --- | --- | --- | --- |
| SRLMQ |  |  |  |  |  |  |
| Forethought phase |  |  |  |  |  |  |
| **Goal setting** | 6 | .82 | 3.48 | .71 | -.14 | -.46 |
| **Self-efficacy** | 4 | .63 | 4.04 | .53 | -.40 | .39 |
| **Time management** | 3 | .62 | 3.06 | .80 | -.07 | -.34 |
| Performance phase |  |  |  |  |  |  |
| *Psychological skills:* |  |  | *3.29* | *.53* | *.11* | *.04* |
| **Arousal-regulation** | 3 | .77 | 3.18 | .98 | -.33 | -.63 |
| **Concentration** | 3 | .79 | 3.23 | .78 | -.08 | -.10 |
| **Self-control** | 3 | .64 | 3.06 | .84 | .03 | -.50 |
| **Self-observation** | 3 | .65 | 3.59 | .73 | -.35 | .27 |
| **Imagery** | 2 | .81 | 3.39 | .96 | -.48 | -.02 |
| Self-reflection phase |  |  |  |  |  |  |
| **Coping** | 3 | .75 | 3.90 | .78 | -.47 | -.28 |
| **Perception of progress** | 1 | - | 3.61 | .98 | -.57 | -.13 |
| **Self-evaluation** | 3 | .62 | 3.18 | .77 | -.12 | -.22 |
| ***Total SRL score*** |  |  | *3.43* | *.45* | *.02* | *.15* |
| ERQ |  |  |  |  |  |  |
| **Reappraisal** | 6 | .85 | 5.01 | .98 | -.58 | .70 |
| **Suppression** | 4 | .77 | 3.55 | 1.23 | .17 | -.53 |
| **Rumination** | 4 | .62 | 5.21 | .90 | -.47 | .36 |
| **Repression** | 5 | .55 | 2.47 | .92 | .81 | .56 |

# APPENDIX 4: Item Total Statistics

| *Table A4.1.* Item-Total Statistics for Self-efficacy | | | | |
| --- | --- | --- | --- | --- |
|  | *Scale Mean if Item Deleted* | *Scale Variance if Item Deleted* | *Corrected Item-Total Correlation* | *Cronbach's Alpha if Item Deleted* |
| Self-efficacy 7 (SRLM) | 12.1946 | 2.764 | .432 | .548 |
| Self-efficacy 8 (SRLM) | 11.9880 | 2.889 | .519 | .497 |
| Self-efficacy 9 (SRLM) | 12.1497 | 2.842 | .471 | .522 |
| Self-efficacy 10 (SRLM) | 12.2066 | 3.029 | .264 | .679 |

| *Table A4.2.* Item-Total Statistics for Time management | | | | |
| --- | --- | --- | --- | --- |
|  | *Scale Mean if Item Deleted* | *Scale Variance if Item Deleted* | *Corrected Item-Total Correlation* | *Cronbach's Alpha if Item Deleted* |
| Time-management 11 (SRLM) | 6.0449 | 2.517 | .474 | .457 |
| Time-management 12 (SRLM) | 6.2246 | 3.682 | .280 | .709 |
| Time-management 13 (SRLM) | 6.1138 | 3.008 | .567 | .342 |

| *Table A4.3.* Item-Total Statistics for Self-observation | | | | |
| --- | --- | --- | --- | --- |
|  | *Scale Mean if Item Deleted* | *Scale Variance if Item Deleted* | *Corrected Item-Total Correlation* | *Cronbach's Alpha if Item Deleted* |
| Self-observation 18 (SRLM) | 7.0778 | 2.739 | .360 | .686 |
| Self-observation 19 (SRLM) | 7.2575 | 2.498 | .442 | .577 |
| Self-observation 20 (SRLM) | 7.1916 | 2.360 | .594 | .372 |

| *Table A5****.*** | Pearson correlation among multiple regression variables. | | | | |  | |  |  |
| --- | --- | --- | --- | --- | --- | --- | --- | --- | --- |
| *Variable* | | Total SRL score | Reappraisal | Suppression | Rumination | | Repression | Practice hours | Level of expertise |
| Total SRL score | | **-** |  |  |  | |  |  |  |
| Reappraisal | | **r=.38, *p*<.001** | **-** |  |  | |  |  |  |
| Suppression | | **r=-.12, *p*=.02** | r=.08,  *p=*.07 | **-** |  | |  |  |  |
| Rumination | | r=-.05, *p*=.17 | r=.05,  *p*=.21 | r=-.08,  *p*=.06 | - | |  |  |  |
| Repression | | **r=-.19, *p*<.001** | r=-.06, *p*=.14 | **r=.56, *p*<.001** | **r=-.10, *p*=.03** | | **-** |  |  |
| Practice hours | | **r=.20, *p*<.001** | r=.06,  *p*=.14 | **r=.11, *p*=.03** | r=-.08, *p*=.08 | | **r=.10, *p*=.03** | **-** |  |
| Level of expertise | | **r=.24, *p*<.001** | r=.07,  *p*=.10 | **r=-.16, *p*=.002** | **r=-.10, *p*=.04** | | **r=-.10, *p*=.04** | **r=-.13, *p*=.01** | **-** |

# APPENDIX 5: Pearson correlation among multiple regression variables to check multicollinearity

# APPENDIX 6: Factorial ANOVA (2x2x2) descriptive and test statistics for all variables

*Table A6.1.* Factorial ANOVA descriptive statistics with ***reappraisal*** as the dependent variable.

| Gender | Main occupation: solo or group music making | Level of musical expertise: student or professional | Mean | Std. Deviation | N |
| --- | --- | --- | --- | --- | --- |
| Female | Group | Student | 4.84 | 1.09 | 62 |
|  |  | Professional | 5.25 | 0.96 | 55 |
|  |  | Total | 5.03 | 1.05 | 117 |
|  | Solo | Student | 4.90 | 0.90 | 62 |
|  |  | Professional | 5.08 | 1.00 | 36 |
|  |  | Total | 4.97 | 0.94 | 98 |
|  | Total | Student | 4.87 | 1.00 | 124 |
|  |  | Professional | 5.19 | 0.98 | 91 |
|  |  | Total | 5.00 | 1.00 | 215 |
| Male | Group | Student | 4.98 | 0.84 | 26 |
|  |  | Professional | 4.91 | 1.00 | 27 |
|  |  | Total | 4.95 | 0.92 | 53 |
|  | Solo | Student | 5.23 | 0.84 | 37 |
|  |  | Professional | 4.95 | 1.08 | 29 |
|  |  | Total | 5.10 | 0.96 | 66 |
|  | Total | Student | 5.12 | 0.84 | 63 |
|  |  | Professional | 4.93 | 1.03 | 56 |
|  |  | Total | 5.03 | 0.94 | 119 |
| Total | Group | Student | 4.88 | 1.02 | 88 |
|  |  | Professional | 5.14 | 0.98 | 82 |
|  |  | Total | 5.01 | 1.01 | 170 |
|  | Solo | Student | 5.02 | 0.89 | 99 |
|  |  | Professional | 5.02 | 1.03 | 65 |
|  |  | Total | 5.02 | 0.95 | 164 |
|  | Total | Student | 4.95 | 0.95 | 187 |
|  |  | Professional | 5.09 | 1.00 | 147 |
|  |  | Total | 5.01 | 0.98 | 334 |

*Table A6.2.* Factorial ANOVA Tests of Between-Subjects Effects with ***reappraisal*** as the dependent variable.

| Groups | *F* | *p* | Partial Eta Squared |
| --- | --- | --- | --- |
| Gender | .000 | .998 | .000 |
| Level of musical expertise | .324 | .570 | .001 |
| Main occupation | .146 | .702 | .000 |
| Gender * Level of musical expertise | 4.382 | .037 | .013 |
| Gender * Main occupation | .729 | .394 | .002 |
| Level of musical expertise * Main occupation | .949 | .331 | .003 |
| Gender * Level of musical expertise * Main occupation | .002 | .964 | .000 |

| *Table A6.3.* Factorial ANOVA descriptive statistics with ***suppression*** as the dependent variable. | | | | | |
| --- | --- | --- | --- | --- | --- |
| Gender | Main occupation: solo or group music making | Level of musical activity: student or professional | Mean | Std. Deviation | N |
| Female | Group | Student | 3.66 | 1.26 | 62 |
|  |  | Professional | 3.22 | 1.26 | 55 |
|  |  | Total | 3.45 | 1.27 | 117 |
|  | Solo | Student | 3.63 | 1.120 | 62 |
|  |  | Professional | 3.35 | 1.11 | 36 |
|  |  | Total | 3.53 | 1.17 | 98 |
|  | Total | Student | 3.64 | 1.22 | 124 |
|  |  | Professional | 3.28 | 1.19 | 91 |
|  |  | Total | 3.49 | 1.22 | 215 |
| Male | Group | Student | 3.94 | 1.40 | 26 |
|  |  | Professional | 3.49 | 1.09 | 27 |
|  |  | Total | 3.71 | 1.26 | 53 |
|  | Solo | Student | 3.80 | 1.32 | 37 |
|  |  | Professional | 3.37 | 1.07 | 29 |
|  |  | Total | 3.61 | 1.22 | 66 |
|  | Total | Student | 3.86 | 1.34 | 63 |
|  |  | Professional | 3.43 | 1.07 | 56 |
|  |  | Total | 3.66 | 1.24 | 119 |
| Total | Group | Student | 3.74 | 1.30 | 88 |
|  |  | Professional | 3.31 | 1.20 | 82 |
|  |  | Total | 3.53 | 1.27 | 170 |
|  | Solo | Student | 3.69 | 1.24 | 99 |
|  |  | Professional | 3.36 | 1.08 | 65 |
|  |  | Total | 3.56 | 1.19 | 164 |
|  | Total | Student | 3.72 | 1.27 | 187 |
|  |  | Professional | 3.33 | 1.15 | 147 |
|  |  | Total | 3.55 | 1.23 | 334 |

*Table A6.4.* Factorial ANOVA Tests of Between-Subjects Effects with ***suppression*** as the dependent variable.

| Groups | *F* | *p* | Partial Eta Squared |
| --- | --- | --- | --- |
| Gender | 1.695 | .194 | .005 |
| Level of musical expertise | 7.849 | .005 | .024 |
| Main occupation | .082 | .775 | .000 |
| Gender * Level of musical expertise | .089 | .766 | .000 |
| Gender * Main occupation | .422 | .516 | .001 |
| Level of musical expertise * Main occupation | .106 | .745 | .000 |
| Gender * Level of musical expertise * Main occupation | .057 | .812 | .000 |

| *Table A6.5.* Factorial ANOVA descriptive statistics with ***repression*** as the dependent variable. | | | | | |
| --- | --- | --- | --- | --- | --- |
| Gender | Main occupation: solo or group music making | Level of musical activity: student or professional | Mean | Std. Deviation | N |
| Female | Group | Student | 2.72 | 1.08 | 62 |
|  |  | Professional | 2.18 | 0.86 | 55 |
|  |  | Total | 2.47 | 1.02 | 117 |
|  | Solo | Student | 2.49 | 0.78 | 62 |
|  |  | Professional | 2.13 | 0.68 | 36 |
|  |  | Total | 2.36 | 0.76 | 98 |
|  | Total | Student | 2.60 | 0.95 | 124 |
|  |  | Professional | 2.16 | 0.79 | 91 |
|  |  | Total | 2.42 | 0.91 | 215 |
| Male | Group | Student | 2.79 | 1.09 | 26 |
|  |  | Professional | 2.28 | 0.79 | 27 |
|  |  | Total | 2.53 | 0.97 | 53 |
|  | Solo | Student | 2.71 | 0.96 | 37 |
|  |  | Professional | 2.42 | 0.86 | 29 |
|  |  | Total | 2.59 | 0.93 | 66 |
|  | Total | Student | 2.74 | 1.01 | 63 |
|  |  | Professional | 2.35 | 0.82 | 56 |
|  |  | Total | 2.56 | 0.94 | 119 |
| Total | Group | Student | 2.74 | 1.08 | 88 |
|  |  | Professional | 2.22 | 0.83 | 82 |
|  |  | Total | 2.49 | 1.00 | 170 |
|  | Solo | Student | 2.57 | 0.85 | 99 |
|  |  | Professional | 2.26 | 0.78 | 65 |
|  |  | Total | 2.45 | 0.84 | 164 |
|  | Total | Student | 2.65 | 0.97 | 187 |
|  |  | Professional | 2.23 | 0.81 | 147 |
|  |  | Total | 2.47 | 0.92 | 334 |

*Table A6.6.* Factorial ANOVA Tests of Between-Subjects Effects with ***repression*** as the dependent variable.

| Groups | F | *p* | Partial Eta Squared |
| --- | --- | --- | --- |
| Gender | 2.682 | .102 | .008 |
| Level of musical expertise | 16.414 | .000 | .048 |
| Main occupation | .273 | .602 | .001 |
| Gender * Level of musical expertise | .058 | .809 | .000 |
| Gender * Main occupation | .719 | .397 | .002 |
| Level of musical expertise * Main occupation | .865 | .353 | .003 |
| Gender * Level of musical expertise * Main occupation | .006 | .939 | .000 |

| *Table A6.7***.** Factorial ANOVA descriptive statistics with ***rumination*** as the dependent variable. | | | | | |
| --- | --- | --- | --- | --- | --- |
| Gender | Main occupation: solo or group music making | Level of musical activity: student or professional | Mean | Std. Deviation | N |
| Female | Group | Student | 5.50 | 0.87 | 62 |
|  |  | Professional | 5.32 | 0.82 | 55 |
|  |  | Total | 5.42 | 0.85 | 117 |
|  | Solo | Student | 5.30 | 0.79 | 62 |
|  |  | Professional | 5.04 | 1.01 | 36 |
|  |  | Total | 5.21 | 0.88 | 98 |
|  | Total | Student | 5.40 | 0.84 | 124 |
|  |  | Professional | 5.21 | 0.90 | 91 |
|  |  | Total | 5.32 | 0.87 | 215 |
| Male | Group | Student | 4.78 | 0.92 | 26 |
|  |  | Professional | 4.74 | 1.08 | 27 |
|  |  | Total | 4.76 | 1.00 | 53 |
|  | Solo | Student | 5.26 | 0.90 | 37 |
|  |  | Professional | 5.13 | 0.76 | 29 |
|  |  | Total | 5.20 | 0.84 | 66 |
|  | Total | Student | 5.06 | 0.93 | 63 |
|  |  | Professional | 4.94 | 0.94 | 56 |
|  |  | Total | 5.01 | 0.93 | 119 |
| Total | Group | Student | 5.29 | 0.94 | 88 |
|  |  | Professional | 5.13 | 0.95 | 82 |
|  |  | Total | 5.21 | 0.95 | 170 |
|  | Solo | Student | 5.29 | 0.83 | 99 |
|  |  | Professional | 5.08 | 0.90 | 65 |
|  |  | Total | 5.21 | 0.86 | 164 |
|  | Total | Student | 5.29 | 0.88 | 187 |
|  |  | Professional | 5.11 | 0.92 | 147 |
|  |  | Total | 5.21 | 0.90 | 334 |

*Table A6.8.* Factorial ANOVA Tests of Between-Subjects Effects with ***rumination*** as the dependent variable.

| Groups | *F* | *p* | Partial Eta Squared |
| --- | --- | --- | --- |
| Gender | 9.399 | .002 | .028 |
| Level of musical expertise | 2.290 | .131 | .007 |
| Main occupation | .933 | .335 | .003 |
| Gender * Level of musical expertise | .450 | .503 | .001 |
| Gender * Main occupation | 10.917 | .001 | .032 |
| Level of musical expertise * Main occupation | .175 | .676 | .001 |
| Gender * Level of musical expertise * Main occupation | .003 | .959 | .000 |
